# Entero-vascular fistula following radiotherapy in a patient with recurrent cervical cancer post-pelvic exenteration: a case report

**DOI:** 10.1186/s12905-025-03840-x

**Published:** 2025-07-09

**Authors:** In Sun Hwang, Soo Young Hur

**Affiliations:** Department of Obstetrics and Gynecology, Seoul St. Mary’s Hospital, College of Medicine, The Catholic University of Korea, 222, Banpo-daero, Seocho-gu, Seoul, 06591 Republic of Korea

**Keywords:** Pelvic exenteration, Uterine cervical neoplasms, Chemoradiotherapy, Palliative care, Vascular fistula

## Abstract

**Background:**

Total pelvic exenteration (PE) is a surgical resection of all pelvic organs used as a palliative treatment for locally advanced or recurrent pelvic malignancies. This case report describes an entero-vascular fistula as a severe complication following radiotherapy in a patient with recurrent cervical cancer who underwent PE.

**Case presentation:**

We present the case of a 47-year-old woman who was diagnosed with cervical cancer at the age of 43 years, classified as FIGO stage IIB. She underwent a radical hysterectomy followed by concurrent chemoradiotherapy (CCRT). In 2023, she developed a recto-urethral fistula and subsequently underwent PE. In March 2024, she was admitted for right buttock and leg pain. An evaluation revealed bone metastasis in the lumbar vertebrae, left iliac bone, and sacrum, for which local radiotherapy was administered. Later, she complained of bloody discharge from a sacral fistula and upper abdominal pain. Her blood pressure was 105/65 mmHg, heart rate 75 beats per minute (BPM), and hemoglobin level was 7.8 g/dL, prompting an emergency blood transfusion. Abdomino-pelvic computed tomography (APCT) scan and esophagogastroduodenoscopy (EGD) revealed a large amount of bloody fluid in the stomach and suggested communication with the bowel loop. CT angiography showed contrast extravasation from the left external iliac artery and a large hematoma. A stent graft was inserted at the site of the entero-vascular fistula to achieve embolization.

**Conclusion:**

This case highlights an entero-vascular fistula as one of the severe complications following PE and radiotherapy for recurrent cervical cancer. Patients who have undergone multimodal treatment, including PE, may have a pelvic condition that is more vulnerable to radiation. Therefore, the complications that may arise from radiation therapy, such as fistula formation, could be higher compared to patients who have not undergone PE.

## Background

Total pelvic exenteration (PE) is a surgical resection of all pelvic organs, including the rectum, bladder, and the reproductive organs, for locally advanced primary or recurrent pelvic malignancies [1]. This radical surgery is often the last curative option for patients who have undergone multimodal treatment. Traditionally, this aggressive surgical extirpation has been associated with a high rate of complications and few long-term survivors [2]. However, since Brunschwig first described it in 1948 for the treatment of recurrent gynecologic malignancies [3], advances in surgical techniques and perioperative care have significantly decreased surgical morbidity and mortality rates [1, 4].

Five-year survival rates after PE have been reported to range between 28.2 and 59% with mortality rates approximately 0.5-2% [1, 5, 6]. The 30-day morbidity rate was reported to be 67.2%, often associated with three or more major complications [7]. Commonly reported major peri- or post-operative complications include rectovaginal fistula, anastomotic site leak, sepsis, and pulmonary embolism.

PE is considered a palliative surgical treatment for advanced or recurrent cervical cancer and is worthwhile when the expected survival is approximately 1 year [8, 9]. While PE remains the final curative option in select patients, recent advances in systemic therapies — such as immune checkpoint inhibitors [10], targeted therapies [11], and image-guided brachytherapy [12] — have expanded the therapeutic landscape for recurrent or metastatic cervical cancer, Nevertherless, in paitens with central pelvic recurrence confined to the pelvis, surgical resection may still offer the only chance of cure.

This case report describes a patient with recurrent cervical cancer who developed bone metastasis following PE and subsequently experienced an entero-vascular fistula as a severe complication during radiotherapy.

## Case presentation

We present the case of a 47-year-old woman who was diagnosed at 43 years of age in 2020 with cervical cancer, specifically mucinous carcinoma of the gastric type, well-differentiated, involving the parametrium, and classified as FIGO stage IIB. She underwent a radical hysterectomy followed by concurrent chemoradiotherapy (CCRT). In 2023, due to the development of a recto-urethral fistula, she subsequently underwent PE, including abdominoperineal resection (APR), right hemicolectomy, total vaginectomy, radical cystectomy with ileal conduit, ureterostomy, and multiple tumor excisions around the left external iliac artery and the right common iliac artery.

The patient presented to the emergency room in March 2024 with a chief complaint of right buttock and leg pain. She was admitted for evaluation and pain control. Magnetic resonance imaging (MRI) and positron emission tomography (PET) scans revealed bone metastasis in the third and fifth lumbar vertebrae (L3-L5), left iliac bone, and sacrum. Local radiotherapy was administered.

On the 36th day of hospitalization, the patient complained of bloody discharge from a sacral fistula. A 5 mm-sized fistula near the sacrum was identified, from which bloody stool was noted, even though the patient had a colostomy that produced normal stool. Her blood pressure was 105/65 mmHg with a heart rate of 75 beats per minute, and her hemoglobin level was 7.8 g/dL, prompting an emergency blood transfusion.

**Fig. 1:**
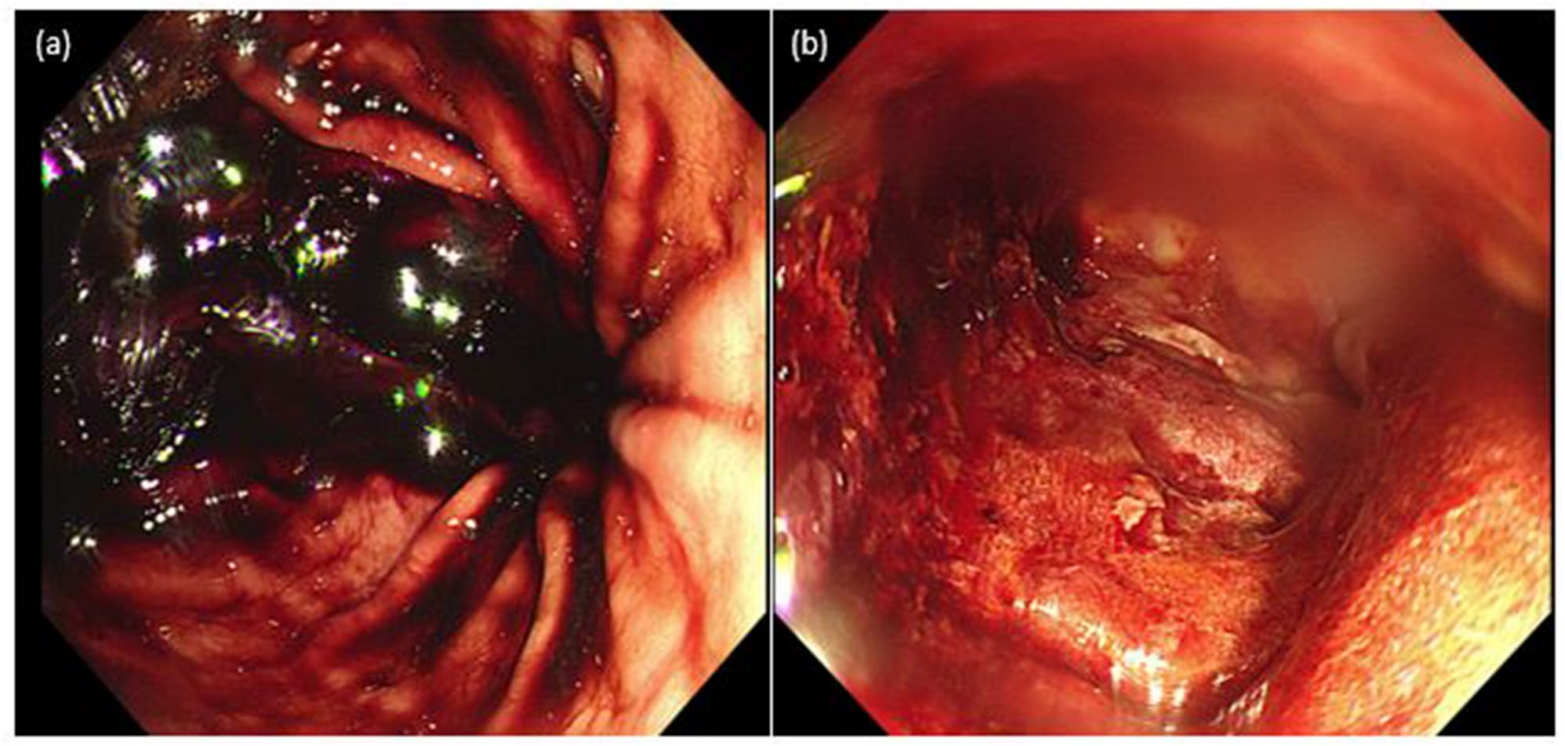
Endoscopic findings indicating ongoing gastrointestinal bleeding. (**a**) Blood pooling was observed in the stomach on immediate esophagogastroduodenoscopy (EGD), suggesting upper gastrointestinal bleeding. (**b**) Sigmoidoscopy revealed a large amount of bloody fluid in the pelvic cavity draining through a fistulous opening on the sacrum, indicating a possible communication between the gastrointestinal tract and surrounding vasculature. These findings were suggestive of an entero-vascular fistula as the underlying source of hemorrhage

Simultaneously, the patient complained of cramping pain in the upper abdomen, leading to an emergency abdominopelvic computed tomography (APCT) scan and esophagogastroduodenoscopy (EGD). The APCT showed air bubbles in a complicated fluid collection in the presacral area, suggesting communication with a bowel loop. Immediate EGD demonstrated blood pooling in the stomach, but no active bleeding lesions were found. The yellow stool was observed at the ileostomy site. Examination through the fistula revealed a large amount of bloody fluid collection in the pelvic cavity; however, communication with the bowel loop was difficult to observe (Fig. 1).

**Fig. 2:**
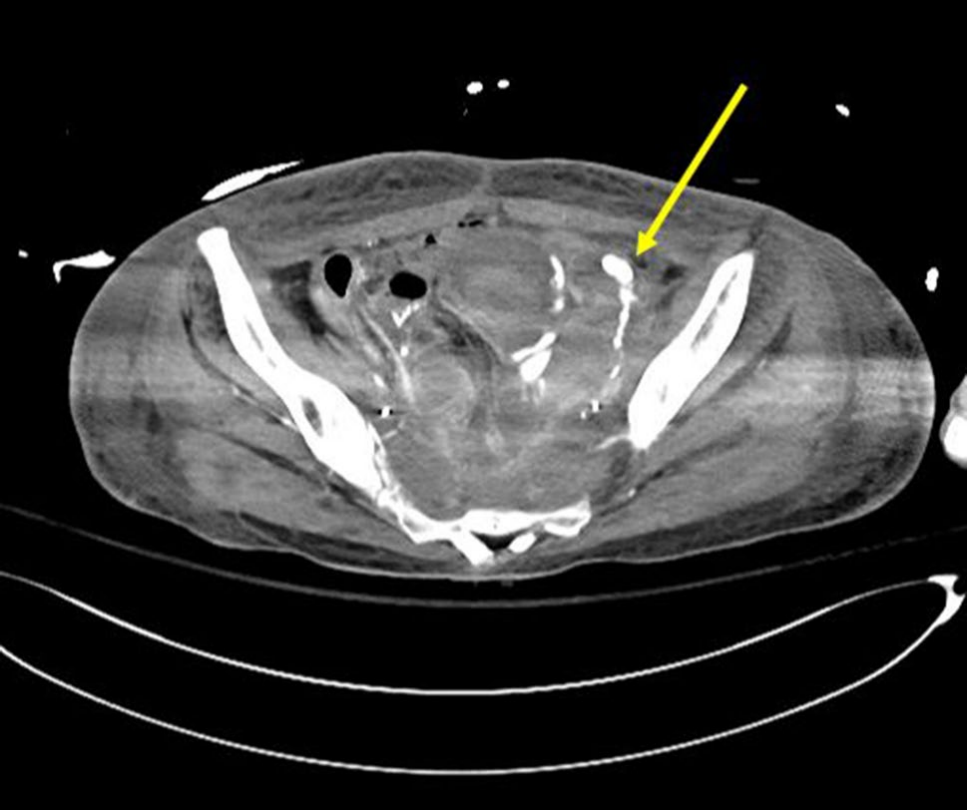
Contrast-enhanced CT angiography showing active extravasation in the pelvic cavity. The arrow indicates the site of contrast leakage, which was interpreted as evidence of ongoing hemorrhage from a vascular source. This finding guided the decision to proceed with embolization, suggesting the presence of an underlying entero-vascular fistula

To identify the active bleeding focus, a CT angiography was performed, which revealed contrast extravasation from the left external iliac artery and a large amount of hematoma. Additionally, contrast extravasation was observed in the adjacent pelvic ileal loop, indicating active bleeding from the left external iliac artery with a pelvic ileal fistula (Fig. 2). Abdominal aortography and left iliac angiography were performed, and a 6 mm–4 cm stent graft was inserted at the site of the arterial rupture to achieve embolization. Despite emergency procedures, including blood transfusions and the use of vasopressors, the patient succumbed to hypovolemic shock later the same day. Her hospital course is summarized as a timeline in Table 1.

**Table 1:** Timeline for the patient’s hospitalization

Patient Information	
Age/Sex	47-year-old female
Diagnosis	Cervical cancer (FIGO stage IIB, diagnosed in 2020)
Treatment History	Radical hysterectomy and adjuvant concurrent chemoradiotherapy (2020) Systemic chemotherapy and salvage radiotherapy (2022) Pelvic exenteration (2023)
Hospital Day	Clinical Course
HD#1	Visited the emergency room in March 2024 with complaints of right buttock and leg pain. Admitted for evaluation and pain control.
HD#10	PET-CT performed.
HD#15	Pelvic MRI performed.
HD#36	A fistula-like opening was observed in the sacrococcygeal skin, with bloody discharge draining from the site. The patient subsequently complained of sudden-onset cramping upper abdominal pain. APCT and EGD were performed.
HD#37	09:00 AM– CT angiography and embolization performed Post-procedure– Temporary stabilization, blood transfusion administered, close monitoring 02:00 PM– Sudden hypotension and loss of consciousness 03:00 PM– Death declared following CPR.

## Discussion

Pelvic exenteration (PE) is a radical procedure primarily used in advanced or recurrent cervical cancer to achieve complete tumor resection. While PE remains one of the few therapeutic options after multimodal treatment failure, it is associated with significant peri-and post-operative morbidity [13]. Reported complication rates range between 32 and 86% [14], with common post-operative issues including wound infection, wound dehiscence, and abdominal or pelvic collections. While less frequent compared to these complications, fistulae occur in approximately 1–2% of cases [15]. The most common type of fistula following PE is enterocutaneous, especially in the perineal region. Other types such as vesicovaginal, rectovaginal, and urinary fistulae have also been reported, but entero-vascular fistulae are extremely rare [16].

Based on a systematic review of fistulae formation in colorectal and bladder cancer cases, entero-vascular fistula is particularly uncommon in these malignancies. Colovesical fistulae, which are more frequently reported, occur in up to 6.5% of cancer cases, whereas entero-vascular fistula remains rarer [17].

Regarding patients with cervical cancer, adverse events related to PE have been reported to range between 25 and 83.3%, with notably higher incidences in patients who have previously received radiation therapy [18]. Many studies have explored surgical techniques and prognostic factors aimed at improving surgical outcomes. Additionally, research has investigated long-term survival outcomes for patients undergoing PE for gynecological malignancies.

A retrospective study conducted in China on patients who underwent PE found that 17 (41.5%) out of 41 patients experienced uncontrolled and recurrent cases [19]. Another retrospective study in Greece analyzed 138 cases of gynecologic cancers treated with PE, reporting a recurrence rate of 54.3%. Notably, patients with recurrent cervical cancer had a higher recurrence rate compared to those with other types of gynecologic cancers, including endometrial and ovarian cancers, although this difference was not statistically significant (*p* = 0.266) [20].

In 2012, the National Cancer Center in Korea reviewed patient characteristics, surgical outcomes, survival, recurrence, and complications in curative PE treatment [21]. The study found that eight patients experienced pelvic and distant recurrences. Subsequent treatments included chemotherapy for one patient, surgery and chemotherapy for one patient, radiation therapy and chemotherapy for seven patients, and hospice management for 12 patients. Eighteen patients died from the disease.

Survival outcome were more strongly associated with R0 than R1 resection. Additionally, surgical outcomes can be influenced by prognostic factors such as lymph node involvement, the presence of perineural invasion, and the number of organs involved [22].

Colorectal cancer, a type of pelvic malignancy eligible for PE, shows improved local control with higher doses of radiotherapy. To achieve this while minimizing harm to healthy tissue, intraoperative radiotherapy (IORT) is used [23]. This approach delivers a high dose of radiation directly to the tumor bed after surgical resection. A large systematic review of 3,003 patients treated with IORT for locally advanced or recurrent rectal cancer demonstrated a significant improvement in local recurrence, disease-free survival and overall survival (HR 0.33; 95% CI 0.2– 0.54 *p* = 0.001) [24]. The overall complication rate ranged between 15 and 59%, with short-term complications reported in 3-46% of cases, predominantly wound-related. Gastrointestinal fistulas have been identified as post-treatment complications, with incidence ranging between 1 and 8%.

This case highlights the rare but severe complications of entero-vascular fistulas following PE and radiotherapy for recurrent cervical cancer. Entero-vascular fistulas are uncommon but life-threatening complications that require prompt diagnosis and management. Our patient’s presentation with bloody discharge from a sacral fistula, despite having a colostomy, underscores the complexity of post-surgical monitoring in these patients. In this case, timely diagnosis was complicated by the absence of visible hematochezia due to the colostomy, which delayed recognition of active gastrointestinal bleeding. Although CT angiography revealed active extravasation and guided embolization, endoscopic evaluation failed to localize the bleeding source, highlighting the diagnostic challenges in such settings. Despite prompt intervention, the patient’s condition deteriorated, underscoring the limited efficacy of embolization alone in managing radiation-induced arterio-enteric fistulas.

The findings suggest that closer monitoring and proactive management strategies are necessary to address potential complications early. Future research should focus on developing predictive models to identify patients at higher risk for such complications and explore innovative techniques to prevent fistula formation.

In conclusion, this case serves as a reminder of the complexities involved in managing advanced pelvic malignancies and the critical role of timely intervention in mitigating severe complications. Clinicians should maintain a high index of suspicion for vascular complications in patients presenting with unusual symptoms post-PE, and a coordinated, multidisciplinary approach is essential for optimal care.

## Conclusions

This case highlights an entero-vascular fistula as one of the severe complications following PE and radiotherapy for recurrent cervical cancer. In comparison to existing literature, this case emphasizes that PE and radiotherapy present a risk of morbidity, such as fistula formation. Therefore, understanding these risks suggests that careful management strategies must be selected for the treatment of recurrences after PE.

## Data Availability

The data supporting the findings of this study are available from the corresponding author upon reasonable request.

## Abbreviations

APCT: Abdomino pelvic computed tomography
APR: Abdominoperineal resection
CCRT: Concurrent chemoradiotherapy
CI: Confidence interval
CT: Computed Tomography
EGD: Esophagogastroduodenoscopy
HR: Hazard ratio
IORT: Intraoperative radiotherapy
MRI: Magnetic resonance imaging
OS: Overall survival
PE: Pelvic exenteration
PET: Positron emission tomography
PFS: Progression-free survival
